# Effects of community-led total sanitation and hygiene implementation on diarrheal diseases prevention in children less than five years of age in South Western Ethiopia: A quasi- experimental study

**DOI:** 10.1371/journal.pone.0265804

**Published:** 2022-04-25

**Authors:** Gedamu Bushen, Hailu Merga, Fasil Tessema

**Affiliations:** 1 MPH in Filed Epidemiology, South West Sewa Health Department, Oromia Region, Waliso, Ethiopia; 2 Department of Epidemiology, Institute of Health, Jimma University, Jimma, Ethiopia; National Research Centre of Egypt, EGYPT

## Abstract

**Background:**

Lack of improved sanitation is the most important contributing factor to diarrheal disease among under-five children in low and middle-income countries. There was no study to identify the effect of Community-Led Total Sanitation and Hygiene intervention on diarrheal diseases in the study area. Hence, this study was designed with the aim of finding the effects of Community-led Total Sanitation and Hygiene implementation for preventing diarrhea among under-five children.

**Methods:**

A community-based Quasi-Experimental study was conducted among a sample of 846 households selected from intervention (kersa) and comparison (mana) districts using the four-stage random cluster-sampling method. A Semi-structured questionnaire was used to collect data. The collected data was cleaned, coded, and entered into EpiData version 3.1 and exported to SPSS version 20 for analysis. Difference-in Difference method with McNemar’s tests was used to compare the prevalence of diarrhea between the intervention and comparison districts, and the significance of change between the pre-test and post-test was declared at p-value less than 0.05 with 95% confidence interval.

**Results:**

The intervention led to decreased diarrhea prevalence [pp = -8.2, 95% CI: -15.9, -0.5], increased latrine ownership [pp = 5.6, 95% CI: 0.5, 10.8], and increased latrine utilization [pp = 10.7, 95% CI: 4.7, 16.6] in intervention district at post-test compared to the baseline; while the presence of handwashing facility near the latrine, home-based water treatment, and proper water storage and handling practice were decreased at post-test compared to the baseline.

**Conclusion:**

Implementation of Community-Led Total Sanitation improved sanitation and hygiene status of community that resulted in the reduction of diarrhea diseases in under-five children. Further implementation, evaluation, and scale-up of the interventions are needed to reduce diarrheal disease in under-five children.

## Introduction

Diarrhea remains a major public health problem especially in developing countries where it is a leading cause of childhood morbidity and mortality [1]. Although diarrhea has a low prevalence in high-income countries, it can occur in vulnerable populations anywhere [2]. The provision of safe drinking water and sanitation might significantly reduce the hundreds of thousands of deaths caused by diarrheal diseases each year [3].

The use of improved sanitation and excreta disposal were significantly associated with lower odds of diarrhea [4]. An estimated 85% of diarrhea mortality is attributed to unsafe drinking water, inadequate sanitation, and substandard hygiene practices [5]. The practice of open defecation is thought to be a major cause of the persistent worldwide burden of diarrhea and enteric parasite infection among children less than 5 years. Reducing open defecation requires access to and use of improved sanitation facilities, which are defined as facilities that prevent human feces from re-entering the environment [6].

In Ethiopia, about 13% of children under age of 5 years from a nomadic community had diarrhea and diarrhea was responsible for 24–30% of all infant deaths and 25% of mortality among children aged 1 to 4 years [7].

Community Led Total Sanitation (CLTS) is an approach that focuses on sustained behavioral change through motivation and mobilization to facilitate and enhance community knowledge and understanding of the risks associated with open defecation. In Ethiopia, CLTS was the precursor to CLTSH (Community Led Total Sanitation and Hygiene), a modified version that has an added hygiene component. As with its predecessor, CLTSH functions without subsidies and has as its primary goal the achievement of open defecation free (ODF) status in all villages of the country. The approach is aimed at empowering the community to analyze the extent and risks of environmental pollution caused by open defecation and to construct and use toilets with their own resources [8].

Community-Led Total Sanitation is a sanitation promotion based on stimulating collective action to address open defecation and its negative impacts on the entire community. The basic assumption is that no human being can stay unmoved once they have learned that they are ingesting other people’s feces. Generally communities react strongly and immediately try to find ways to change this through their own effort based on different motivations [9].

Observational studies conducted to evaluate the effects of interventions that prevent human feces from entering the environment have shown that they reduce diarrheal diseases and enteric parasite infections [10–15]. Most of those researches, however, have focused on the construction and utilization of household-level latrines. Few studies have been conducted in rural areas of low-income countries where CLTSH intervention is implemented [16, 17].

Latrine ownership is one of the most important variables influenced by a variety of behavioral, cultural, social, geographic, and economic factors among the community. Since both our study districts are rural districts, hygiene and environmental sanitation are particularly poor in rural communities. Almost all respondents were farmers, and inadequate income to purchase latrine facilities, as well as a lack of water, had a significant impact on the prevalence of diarrhea.

There was a WaSH Programming in both intervention and comparison districts. Federal Ministry of Health was implementing the CLTS in some districts only as a pilot study since 2012. Accordingly, during the study, CLTS was implemented only in the intervention district; not in the comparison district. Since all of the kebeles (smallest administrative unit in Ethiopia) have declared open defecation free in Kersa district (intervention district), and there was no finding on the effect of CLTSH intervention in the district, a strong need exists for evidence of the effects of implementation of CLTSH approach in the study area. This study was, therefore, designed with the aim of finding evidence for the effect of implementation of CLTSH approach on the prevalence of diarrheal diseases in under five children.

## Materials and methods

### Study setting

A Quasi-experimental study was conducted in Kersa and Manna districts, Jimma Zone, Oromia regional state, Southwestern Ethiopia, from March 01 to April 10, 2019. These two districts were chosen at random sinvce they are located in the same zone (Jimma Zone administration) and have a comparable population. These populations share common characteristics like language, culture, income, health seeking behaviors, disease prevention, etc., and hence they are comparable population.

Kersa district is found about 370 kilometers away from the capital city, Addis Ababa in the west direction. The district is bounded by Dedo district to the South, Seka Chekorsa district to the Southwest, Manna district to the West, Limu Kosa to the North, Tiro Afeta to the Northeast, and Omo Nada district to the Southeast.

**Fig 1:**
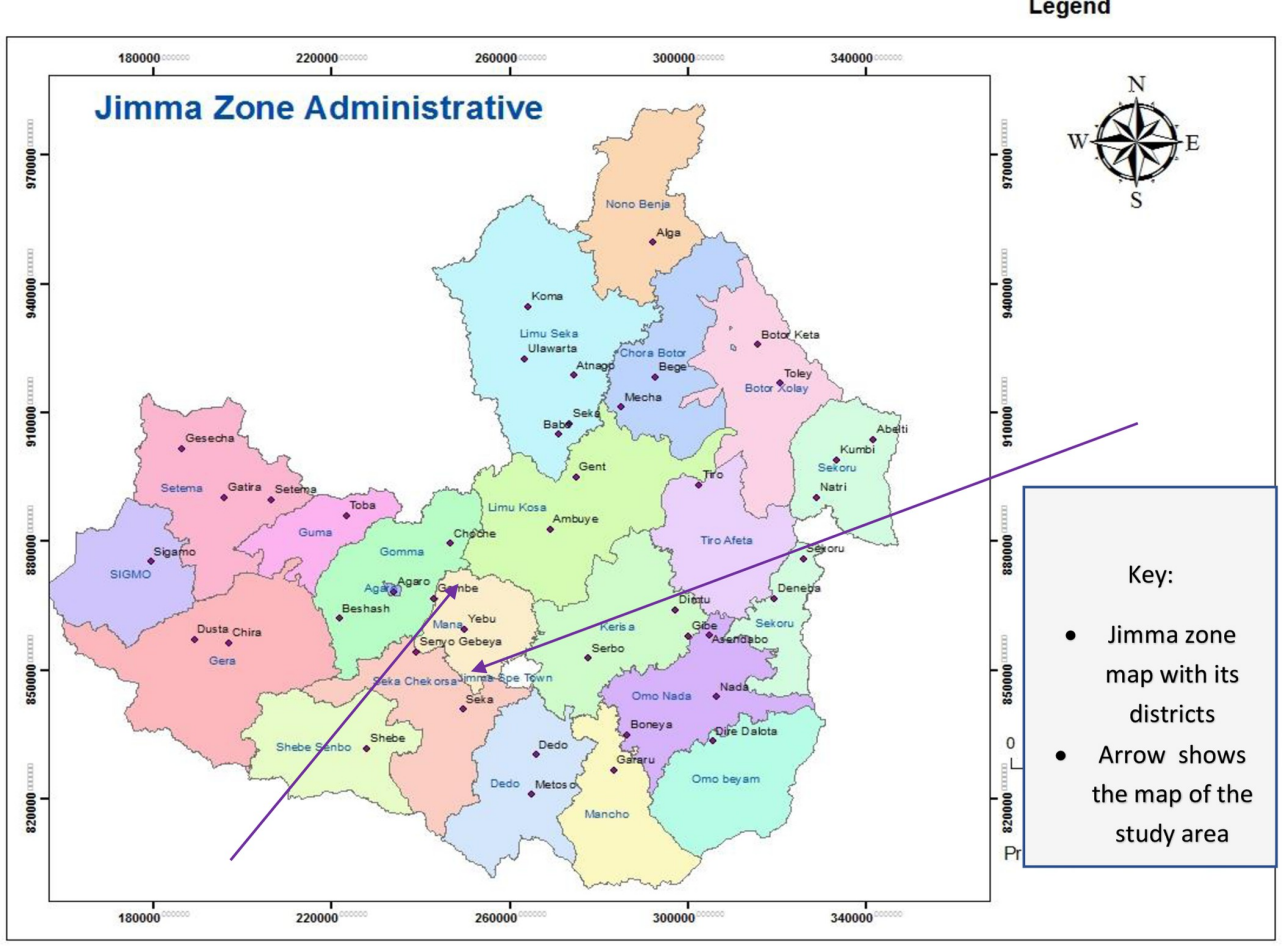
Map of the study area.

Manna district is found about 372 kilometers away from Addis Ababa, in the west direction, on the main asphalt road from Jimma to Agaro, which crosses several districts in the Jimma zone. The district is bordered on the south by Seka chekorsa district, on the north by Limmu Kossa district, on the West by Gomma district, and on the East by Kersa district. According to the 2018 population projections [18], in 2019 Kersa district population is estimated to be 227,803 with male to female ratio of 1:0.98. The average size of a household is estimated to be 4.8. The number of under-five children was 37,428 (16.4%). Similarly, the 2019 projected population of Manna district was 201,992, with male to female ratio of 1:0.96. The average household size is estimated to be 4.8 and the number of under-five children was 33, 187 (16.43%) (Fig 1).

### Population and sampling

The source populations were all households residing in Kersa and Manna districts during the data collection period and the study populations were selected households having children less than five years of age who were residents of the two districts during the data collection period. Households with at least one child under 5 years of age were eligible for this study. The age of a child was verified with an immunization card, which shows the birth date of the child. Households with under-five children, but who were unable to communicate (in the absence of appropriate respondents or when respondents were unable to hear or speak) during data collection were excluded from the study.

The required sample size was calculated using G*Power 3.1.9.2 software considering the following assumptions: 5% for type I error, 80% power, Odds ratio of 2, the proportion of discordant pairs 0.255, the prevalence of diarrhea from a previous study 22.22% [11], the effect size of 10%, and design effect of 1.6. Finally, after adding 10% non-response rate, the total sample size was calculated to be 814 participants (407 for intervention district, and 407 for comparison district).

**Fig 2:**
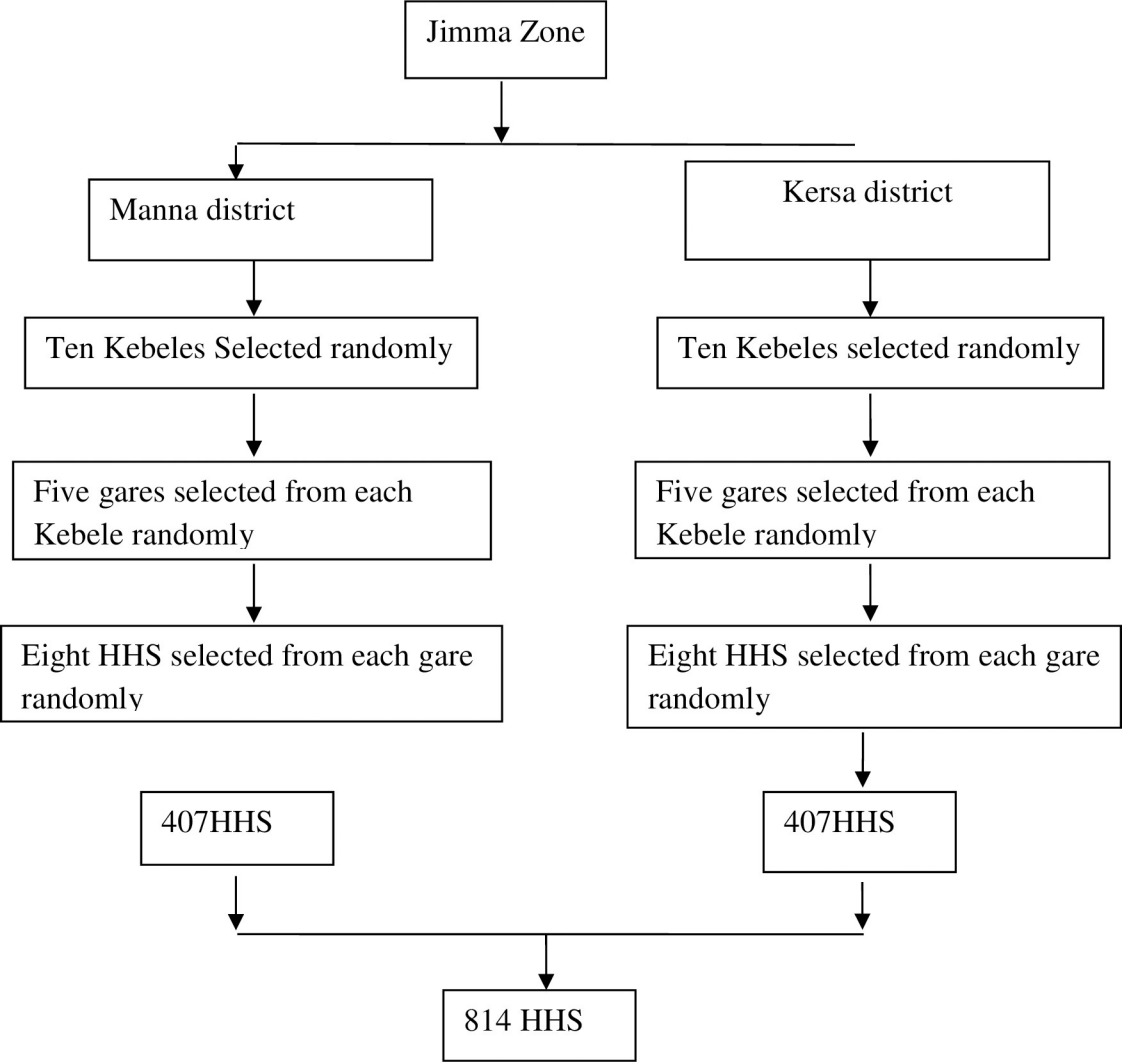
Schematic presentation of sampling procedure.

A four-stage random cluster-sampling technique was employed for selecting the study participants. In the first stage, Kersa and Manna districts were purposively selected. The implementation of CLTSH intervention was used as criteria for selecting and including districts in the study. Accordingly, Kersa district was selected as an intervention district. Simultaneously, non-intervention (Manna district) was selected as a comparison group for comparison. In the second stage, 10 out of the 30 kebeles in the intervention and 10 out of 19 kebeles of the non-intervention district were selected randomly and included in the study (Fig 2).

In the third stage, five “gares” (sub Kebele) were selected from each kebele by using probability proportional to population size (PPS) method. In the fourth stage, eight households were randomly selected from each selected “gare”. The number of households in each Kebele was decided based on the district level household proportion. In the selected households, the youngest child among the children under 5 years old in the household was selected to be included in the study.

### Data collection tool and procedures

#### Baseline data

There was a WaSH Programming in both intervention and comparison districts in the regular activities of each district. However, during the study CLTS was implemented only in the intervention districts; not in the comparison districts. Since we didn`t get appropriate baseline data, we reconstructed the baseline data using secondary data sources (i.e. survey and annual report). To reconstruct the baseline data, we used the report of a study conducted from December 2012 to January 2013 to evaluate the implementation of a Community-led Total Sanitation and Hygiene Approach on the Prevention of Diarrheal Disease in Kersa District, one of the districts in the study area (11) and Jimma Zone health department and study site monthly reports. Majority of the study variables were taken from this study and the health sector report in the district was used to check certain WaSH related indicators.

#### Post-intervention data

Post-intervention data was collected from March 01-April 10, 2019. A semi-structured questionnaire was used to collect data. The data collection tool was adapted from various similar studies [19–21], and modified according to local context (S1 Text). Data collection tool was translated from English to Afan Oromo language, and then back to English to check for consistency by different professionals.

The questionnaire was pre-tested for one day before the actual data collection took place in a non-study area (Omo nada and Seka chekorsa districts) by taking 5% of the sample size and the necessary corrections were made before the actual data collection.

Data were collected using face-to-face interviews of the primary caretaker of the selected child and observation of sanitation facilities and household conditions to assess indicators of behavioral, environmental, and socio-demographic factors at the home level. Before declaring non-respondent households, up to three visits were made at different times by data collectors. There were 8 data collectors and 4 supervisors who were residents of kebeles other than the study area to minimize social desirability bias and with the educational level of secondary school and above. To assure the quality of data, a one-day training was given for data collectors and supervisors on the objective of the study, the methods of data collection, how to recruit eligible households, with practical exercises. The supervisors closely monitored the entire data collection process. Completed questionnaires were collected and delivered to supervisors after checking for consistency and completeness on daily basis. Missing values and outliers were checked before analysis by running descriptive analysis.

### Data analysis

Data were entered into Epi-data version 3.1 software and exported to SPSS Version 20 software for analysis. Descriptive statistics were calculated from household surveys and observations and presented by frequency distribution, summary measures, tables, and graphs. McNemar test (cross-tabulations) and Generalized Linear Model (GLM) analysis were conducted to estimate the prevalence of primary and secondary outcome variables between intervention and comparison districts at baseline and posttest survey. The primary outcome was diarrheal disease prevalence among children less than five years of age, with the household as the unit of analysis. The secondary outcomes assessed were the availability of latrine, latrine utilization, availability of cover for latrine seat, availability of handwashing facility near latrine, availability of soap at handwashing, safe disposal of child feces, solid waste management, hand washing practice at critical times, presence of feces in the compound, and water storage and handling practice. Self-reported latrine use was validated by observation of latrines. Latrines that were full, collapsed structures, or had unstable flooring were categorized as open defecation. The prevalence of primary outcome between baseline and posttest surveys both in the intervention and comparison districts was compared using a Difference-in-Difference estimator. In addition, McNemar’s test was used to compare the status of secondary outcomes between baseline and posttest surveys. Difference in baseline and posttest surveys was declared statistically significant with P-value less than 0.05.

### Ethical approval

Ethical clearance was obtained from the Ethical Review Committee of Institute of Health, Jimma University. A permission letter was also obtained from Jimma zonal health department and respective District Health Offices to conduct the study. In addition, Kebele Administrators were informed about the purpose of the study. Before the commencement of data collection, caregivers of a child (parents or guardians 18 years and above) were well informed by data collectors about the objectives and importance of their involvement in the study, and the confidentiality of the information they provide. Finally, after taking verbal informed consent, caregivers (parents or guardians) of a child who were willing to take part in the study were interviewed. The ethics committees had approved the verbal consent procedure. Code numbers were used in place of identifiers to maintain the confidentiality of participants’ information.

## Results

### Socio-demographic characteristics

Samples of 423 households were included in this study each from intervention and comparison districts. Of all households interviewed, the response rate was 99.5% and 98.1% in intervention and comparison districts, respectively. The majority of respondents in intervention and comparison districts (63.9% and 74.7% respectively), were married. More than 75% of respondents in both intervention and comparison districts were in the age range of 26–40 years. In both districts, the family size of more than half of households was in the range of 3–5. More than 60% of households in both districts had one child under five years of age. On average, almost half of the heads of the households in both intervention and comparison districts were illiterate. The majority of households in both intervention and comparison districts (96.4% and 89.9% respectively), were Oromo by ethnicity. About 70% of heads of households in both intervention and comparison districts were farmers. Regarding their religion, more than 90% of them were Muslims in both intervention and comparison districts. The average monthly income of about 40% of households in both intervention and comparison districts were 350–550 Ethiopian Birr (Table 1).

**Table 1:** Socio-demographic characteristics of study participants in the study districts, Jimma Zone, Oromia Region, Ethiopia, 2019 (N = 836).

Demographic Characteristics	Intervention district (n = 421)		Comparison district (n = 415)	
Status of respondents	Pre-test n (%)	Post-test n (%)	Pre-test n (%)	Post-test n (%)
Husband	185 (43.7)	123 (29.2)	185 (43.7)	87 (21)
Wife	134 (31.7)	269 (63.9)	134 (31.7)	310 (74.7)
Others	104 (24.6)	29 (6.9)	104 (24.6)	18 (4.3)
Age of respondents				
18–25	168 (39.7)	60 (14.3)	168 (39.7)	66 (15.9)
26–40	186 (44)	316 (75.1)	186 (44)	314 (75.7)
> = 41	69 (16.3)	45 (10.7)	69 (16.3)	35 (8.4)
Family size				
<3	97 (22.9)	8 (1.9)	97 (22.9)	5 (1.2)
3–5	177 (41.8)	218 (51.8)	177 (41.8)	237 (57.1)
> = 6	149 (35.2)	195 (46.3)	149 (35.2)	173 (41.7)
Number of <5 children				
1	202 (47.8)	255 (60.6)	202 (47.8)	268 (64.6)
2	86 (20.3)	160 (38)	86 (20.3)	144 (34.7)
3	135 (31.9)	6 (1.4)	135 (31.9)	3 (0.7)
Educational level of				
Illiterate	244 (57.7)	250 (59.4)	244 (57.7)	196 (47.2)
Literate	179 (42.3)	171 (40.6)	179 (42.3)	219 (52.8)
Ethnicity				
Oromo	415 (98.1)	406 (96.4)	415 (98.1)	373 (89.9)
Amara	4 (0.9)	4 (1)	4 (0.9)	12 (2.9)
Keffa	4 (0.9)	1 (0.2)	4 (0.9)	6 (1.4)
Other	0 (0)	10 (2.4)	0 (0)	24 (5.8)
Occupation of households				
Gov’t employee	15 (3.5)	15 (3.6)	15 (3.5)	20 (4.8)
Farmer (housewife)	289 (68.3)	289 (68.6)	289 (68.3)	288 (69.4)
Merchant	117 (27.%)	117 (27.8)	117 (27.7)	107 (25.8)
Religion				
Muslim	322 (76.1)	398 (94.5)	322 (76.1)	375 (90.4)
Orthodox	93 (22)	17 (4)	93 (22)	26 (6.3)
Protestant	8 (1.9)	5 (1.2)	8 (1.9)	12 (2.9)
Other	0 (0)	1 (0.2)	0 (0)	2 (0.5)
Monthly income of households				
<350	193 (45.6)	193 (45.8)	193 (45.6)	154 (37.1)
350–550	82 (19.4)	170 (40.4)	82 (19.4)	163 (39.3)
551–750	21 (5)	28 (6.7)	21 (5)	37 (8.9)
>750	127 (30)	30 (7.1)	127 (30)	61 (14.7)

### Effects of the intervention on primary outcome

**Fig 3:**
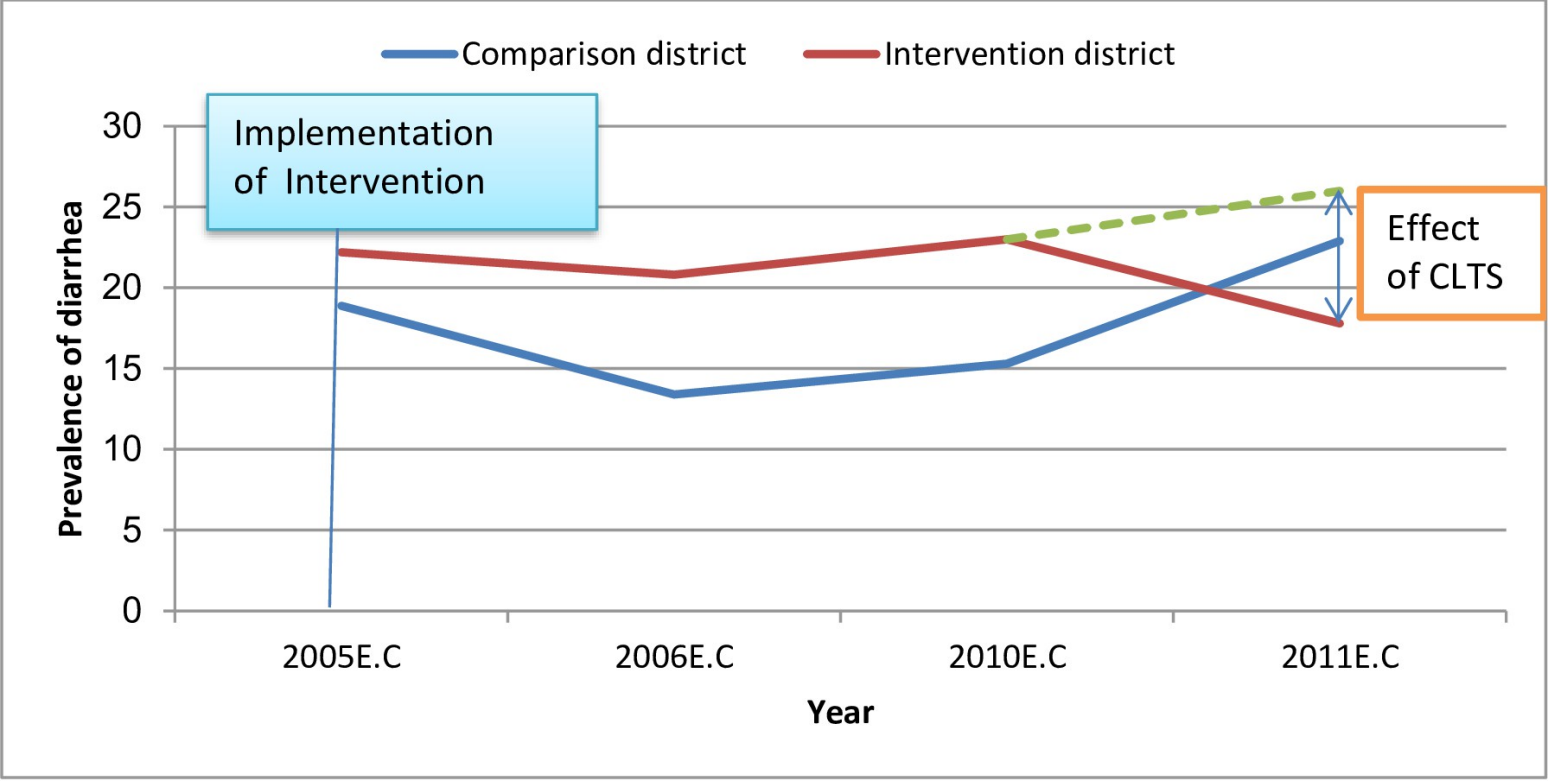
Difference-in-Difference on prevalence of diarrhea in children less than five years in intervention and comparison districts, Jimma zone, Oromia region, southwest Ethiopia, 2019.

Results of Generalized Linear Model (GLM) analysis showed that participants in the intervention district were more likely to report a significant decline in prevalence of diarrhea during the pre-test and post-test periods than those in comparison district [pp = -8.2, 95% CI: -15.9, -0.5] (Table 2 and Fig 3).

**Table 2:** Difference-in-Difference on prevalence of diarrhea in children less than five years in intervention and comparison districts, Jimma zone, Oromia region, Ethiopia, 2019 (N = 836).

District status	Pre-intervention	Post-intervention	Difference with 95% CI)
Intervention district	22.2%	17.8%	-4.4
Comparison district	18.9%	22.7%	3.8
Difference-in-Difference			-4.4–3.8 = -8.2

### Effects of the intervention on secondary outcomes

This study revealed that the intervention had significant effects on several secondary outcome measures. Latrine coverage, availability of cover for latrine seat hole, latrine utilization, availability of handwashing facility near the latrine, availability of soap at hand washing facility, safe management of solid waste, safe disposal of child feces, handwashing practice at critical times, presence of cover for water container, safe drawing of water from the storage, and water treatment at home are expected to increase in intervention district as compared to comparison district, while the presence of feces around the home is expected to decrease in intervention district. Accordingly, the prevalence of secondary outcomes at baseline and post-test for the intervention and comparison districts showed that eight out of twelve indicators suggested differences between intervention and comparison districts in the intended directions.

Compared to households in the comparison district, households in the intervention district were more likely to report increased private ownership of latrine (2.1% and 7.4% point increases respectively) between baseline and posttest periods (p < 0.05). In addition to increased possession of latrine, participants in the intervention district were more likely to report increased latrine utilization as compared to those in comparison district (12.9 and 4.5 percentage point increases respectively) at posttest assessment (p <0.05) compared to baseline. Similarly, participants in the intervention district were more likely to report the availability of latrine seat hole cover (11.6 percentage point increase) at posttest assessment (p <0.05) compared to baseline.

The availability of handwashing facility near latrine was declined in both intervention and comparison districts (decreased by 9.1 and 17.4 percentage points respectively) as compared to the base line, which is the reverse of the intended effect of the intervention. But, the availability of soap at handwashing facility was increased in intervention district, while it was decreased in comparison district as compared to the base line (8.1 percentage point increase and 2.1 percentage point decrease respectively).

Relative to households in comparison district, households in the intervention district reported greater decreases in open defecation in the home surrounding (9.1 and 12.6 percentage points decrease respectively) from baseline to posttest survey. The practice of safe management of solid waste was also increased in intervention district than in comparison district as compared to the base line (20.2 and 11.5 percentage point increases respectively). Similarly, households in the intervention district reported greater increases in safe disposal of child feces from baseline to posttest survey, as compared to comparison district (25.4 and 9.4 percentage point increases respectively).

Participants in both intervention and comparison districts have reported a significant increase on handwashing practice at critical times (12.6 and 12.3 percentage point increases respectively). The availability of cover for water container during storage was declined in intervention district as compared to the base line, which is the reverse of the intended effect of the intervention. Similarly, safe drawing of water from the storage was declined in both intervention and comparison districts as compared to the base line, which is the reverse of the intended effect of the intervention. Home based water treatment was also declined in both intervention and comparison districts as compared to the base line (Tables 3 and 4).

**Table 3:** Prevalence of behavioral and environmental factors associated with diarrheal disease in intervention and comparison districts, Jimma zone, Oromia region, Ethiopia, 2019 (N = 836).

Outcome variables	Intervention district (n = 421)			comparison district (n = 415)		
	Baseline n (%)	Post-test n (%)	p-value	Baseline n (%)	Post-test n (%)	p-value
Latrine coverage	384 (91.2)	415 (98.6)	≤0.0001	365 (88)	374 (90.1)	0.368
Latrine seat hole cover	161 (43.5)	204 (55.1)	0.007	144 (43.6)	166 (50.3)	0.078
Latrine Utilization	317 (85.7)	365 (98.4)	≤0.0001	282 (84.4)	297 (88.9)	0.137
Hand washing facility near latrine	277 (72.7)	239 (62.7)	0.005	242 (72.5)	184 (55.1)	≤0.0001
Soap at hand washing	122 (51)	152 (57.4)	0.271	135 (51.1)	101 (49.3)	0.651
Feces seen around home	86 (20.4)	33 (7.8)	≤0.0001	86 (20.7)	48 (11.6)	0.001
Safe management of solid waste	246 (58.4)	331 (78.6)	≤0.0001	246 (58.4)	294 (70.8)	0.001
Safe disposal of child feces	298 (70.8)	405 (96.2)	≤0.0001	292 (70.4)	331 (79.8)	0.001
Hand washing at critical times	367 (87.2)	420 (99.8)	≤0.0001	361 (87)	412 (99.3)	≤0.0001
Presence of cover for water container	417 (98.6)	412 (97.9)	0.227	409 (98.6)	409 (98.6)	1.000
Safe drawing of water from the storage	322 (76.5)	253 (60.1)	≤0.0001	322 (77.6)	299 (72)	0.069
Water treatment at home	216 (51.3)	67 (15.9)	≤0.0001	216 (52)	86 (20.7)	≤0.0001

**Table 4:** Difference in difference of environmental and behavioral factors associated with diarrheal disease in intervention and comparison districts, Jimma zone, Oromia region, Ethiopia, 2019 (N = 836).

Factors	Difference in intervention district (%)	Difference in comparison district (%)	Difference in difference (%)	P -value	95% CI	
					Lower	Upper
Latrine coverage	7.8	2.2	5.6	0.033	0.5	10.8
Latrine seat hole cover	11.2	5.8	5.4	0.284	-4.5	15.5
Latrine Utilization	13.2	2.5	10.7	≤0.0001	4.7	16.6
Hand washing facility near latrine	6.7	-18.1	11.4	0.017	2	20.9
Soap at hand washing	8.1	-2.1	10.2	0.105	-2.1	22.6
Feces seen around home	-12.5	-9	-3.5	0.307	-10.3	3.2
Safe management of solid waste	20.4	12.6	7.8	0.083	-1	16.7
Safe disposal of child feces	28.6	12.1	16.5	≤0.0001	8.9	24.1
Hand washing at critical times	12.5	12	0.5	0.836	-4.1	5.1
Presence of cover for water container	-0.7	0	-0.7	0.574	-3.1	1.7
Safe drawing of water from the storage	-16	-3.9	-12.1	0.006	-20.7	-3.6
Water treatment at home	-35.1	-30.5	-4.6	0.289	-13.2	3.9

## Discussion

This study was conducted to evaluate the effectiveness of the implementation of Community-Led Total Sanitation and Hygiene (CLTSH) approach on the prevention of diarrheal disease in under-five children. Statistical analyses done on the differences of diarrheal disease prevalence and factors associated with diarrhea between intervention and comparison districts were found to be significant.

This study showed that there was 8.2 percentage point greater reduction in the prevalence of diarrhea after the implementation of the intervention in intervention district. This is supported by a study conducted in Hadaleala district, Ethiopia, which showed that human excreta management was associated with childhood diarrheal disease [7].

In our study, in the intervention district, implementation of CLTSH resulted in a 5.6 percentage point increase in household private latrine ownership than in comparison district. This is supported by a cluster-randomized controlled trial conducted on the effect of a community-led sanitation intervention on child diarrhea and child growth in rural Mali, which showed latrine ownership rose more steeply as a result of CLTS; latrine ownership increased by 39 percentage points [22]. Another study conducted on the effect of CLTS on latrine ownership in Mozambique showed that the proportion of people owning latrine is increasing with increasing extend of CLTS-related information and highest in the group of CLTS participation (79%) [23].

The coverage of private latrine ownership is also greater than the finding of a study conducted rural settings of Dangla District, Northwest Ethiopia, which showed that majority of households in both ODF (89.7%) and OD (92.8%) kebeles had a private latrine [24]. The possible explanation for this variation might be due to the difference in the extent of the implementation of WASH intervention or due to differences in the study design employed.

In addition to increased possession of latrine, participants in the intervention district were more likely to report latrine utilization at posttest assessment compared to baseline. This is supported by a study conducted on the effect of community led total sanitation and hygiene approach on improvement of latrine utilization in Laelay Maichew District, North Ethiopia, which showed that implementation of CLTS improves the utilization of latrine by 16.2 percentage points [16].

This study revealed that the availability of handwashing facility near latrine was reduced in both intervention and comparison districts from baseline to posttest survey. It was reduced from 70.3% to 62.7% (-7.6 pp) and from 74% to 54.8% (-19.2 pp) in intervention and comparison districts respectively. The finding is higher than the study from Yaya Gulele district, which showed that more than half of the participants, (54%) in CLTSH implemented and (63%) unimplemented kebeles had no hand-washing facility in or close to the latrine [25]. The possible explanation for this variation might be due to the difference in the extent of the implementation of the intervention or due to differences in the study period.

Our study showed that from baseline to posttest, the availability of soap at handwashing facility was more increased in intervention district than in comparison district as compared to the baseline, which is greater than the study conducted on Community-Level Sanitation Coverage in rural Mali [22]. This variation might be due to the difference in the level of sanitation coverage and geographical variation between the study populations.

The practice of open defecation was more decreased by 3.5 percentage points in the intervention district, at the posttest assessment compared to baseline. This is almost similar with a study conducted to evaluate the sustainability of community-led total sanitation outcomes in Ethiopia and Ghana, which shows that open defecation practice was decreased by 12 and 17 percentage points respectively, in villages receiving CLTS interventions [26].

CLTS processes can precede and lead on to, or occur simultaneously with, improvement of latrine design; the adoption and improvement of hygienic practices; solid waste management; waste water disposal; care; protection and maintenance of drinking water sources; and other environmental measures [27]. Our finding also showed that there was 20.2 percentage point increase in safe management of solid waste in intervention district. This is greater than the finding of Dabat Health and Demographic Surveillance System conducted on Sanitation predictors of childhood morbidities [28]. This variation might be due to the difference in the level of implementation of WASH intervention.

As with open defecation, unsafe disposal of child excreta poses a health risk to anyone living or playing nearby. When left in the open in the yard or direct vicinity of the household, child feces, which may carry a higher pathogen load than adult feces (child feces can be 20 times more dangerous than adult feces [29, 30], pose a particular risk for young children, whose play areas frequently overlap with disposal areas. Safe disposal of children’s feces is therefore at least as important as stopping open defecation [31]. Our findings also showed that 96.2% of households in intervention district practice safe disposal of child feces, and there is a 16.5 percentage point greater increase in safe disposal of child feces from baseline to posttest survey, as compared to comparison district. This is greater than the evidence from Ethiopia using EDHS data, which showed that 4.72% children used latrine for defecation, 27.84% of children’s stools were put/rinsed into latrine, (42.01%) of children’s stools were left in the open/not disposed of, 14.08% of the children’s stools thrown into garbage, and (1.11%) of children’s stools was buried [32]. It is also greater than the finding from Benishangul Gumuz Regional State, North West Ethiopia, which showed 55% of the households disposed children’s’ stool in an improper manner [33]. This variation might be due to the difference in the study period and level of implementation of WASH intervention.

Participants in both intervention and comparison districts reported a significant increase on hand washing practice at critical times. But there is a 0.5 percentage points greater increase in intervention district from baseline to posttest survey, as compared to comparison district. This is greater than the finding of a study in Sheko district, Southwest Ethiopia and Jabithennan district, Northwest Ethiopia, which shows that 61.5% and 72.6% of households practiced hand washing at critical times respectively [34, 35]. The possible explanations for these variations might be due to the difference in the extent of the implementation of the intervention on the study populations and the study period in which the data collection period for our study is recent than that of these studies.

In our study, the availability of cover for water container during storage was 97.9% and 98.6% in intervention and comparison districts, respectively, but declined as compared to the base line. In intervention district, it was decreased by 1.1% points, which is the reverse of the intended effect of the intervention. This is higher than the finding of a study from Mali, which shows that 96% of households had stored water covered at the time of sample collection [12]. Another similar study with our finding from rural Ethiopia, shows that drinking water was stored in the household primarily in a container with a lid (98%) [36].

In our study, the practice of safe drawing of water from the storage was 60.1% and 72% in intervention and comparison districts, respectively, and declined in both intervention and comparison districts as compared to the base line. In intervention district, it was decreased by 16.4 percentage points, which is the reverse of the intended effect of the intervention. This is lower than the finding of a study conducted on Diarrheal status and associated factors in under five years old children in relation to implemented and unimplemented community-led total sanitation and hygiene in Yaya Gulele district, which shows that the practice of safe drawing of water from the storage was 99% and 84% for CLTS implemented and non-implemented districts respectively [25]. This might be happened due to the difference on the awareness level of the study populations.

This study also revealed that, the practice of water treatment at home level was 15.9% and 20.7% in intervention and comparison districts, respectively, and declined as compared to the base line. In intervention district, it was more decreased by 4.6 percentage points than in comparison district, which is the reverse of the intended effect of the intervention. This is higher than the finding of a study conducted on appropriate household water treatment methods in Ethiopia, which shows that the number of households treating their water prior to drinking with any treatment options was 8.0% in 2005, 10.2% in 2011, and 9.4% in 2016 [37]. The difference might be happened due to the difference on the water sources used by the study populations, and difference on the time of data collection. The possible explanation of the reduction of this practice between the baseline and posttest survey in our study might be due to the expansion of safe water supply between the baseline and posttest survey. On the other hands, finding from low-income country, Kenya, revealed that health education at community level like school health education has big impact on the reduction of diarrheal diseases [5]. Similarly, the implementation of innovative community-based health extension programs in Ethiopia, which place a strong emphasis on raising household sanitation and hygiene awareness, has had a significant impact on the reduction of diarrheal diseases [38].

There were some limitations such as lack of random assignment, which is the major limitation of quasi-experimental study. Besides, since the baseline data was not collected prior to the implementation of the intervention for comparison district, there are threats to the internal validity. Moreover, the use of health sector from the intervention district data for baseline in the study area may affect the validity of the study.

## Conclusion

In this study, we presented the effects of implementation of community-led total sanitation and hygiene approach on the prevalence of diarrheal disease in children less than five years of age. Despite the limitations of the present study, including the nature of the study design and reconstructing of baseline data for comparison district, the results identified levels of improvements in sanitation to deliver the expected health benefits within the intervention district. The findings also suggested that the implementation of community-led total sanitation and hygiene approach can be an effective tool to reduce diarrheal disease prevalence in children under the age of 5 years. Hence, the local health authorities need to work on improvement of some indicators as per CLTS guideline. Moreover, provision of refreshment activities to sustain these observed changes as well as to improve the above-mentioned indicators is recommended. More investigation is needed to evaluate this intervention in different settings and Experimental study would also help to provide a stronger evidential base for the sustainability of the intervention’s effect.

## Supporting information

S1 Text Questionnaire for data collection. (PDF)

## Abbreviations

CLTS: Community-Led Total Sanitation
CLTSH: Community-Led Total Sanitation and Hygiene
GLM: Generalized Linear Model
OD: Open Defecation
ODF: Open Defecation Free
SPSS: Statistical Package for Social Science
