# Supplementary material for: Effects of community-led total sanitation and hygiene implementation on diarrheal diseases prevention in children less than five years of age in South Western Ethiopia: A quasi- experimental study
Source: PLoS One. 2022 Apr 25;17(4):e0265804. doi: 10.1371/journal.pone.0265804 (PMC9037915; doi:10.1371/journal.pone.0265804)
Supplement: S1 Text — (PDF) [file pone.0265804.s001.pdf]

Annex 2: English Version questionnaires

Name of Kebele________________ Name of gott (village) ____________________

Code number of the household________

Name of data collector: __________________________signature____________

PART I. SOCIOECONOMIC AND DEMOGRAPHIC CHARACTERISTICS

Code of questions	QUESTIONS	Responses	Skip to
101	Respondent' status (Relation of the respondent to the child)	1. Father<br>2. Mother<br>3. Grand Father<br>4. Grand Mother<br>5. Sister/Brother<br>6. Other	
102	Family`s dwelling area	1. Urban 2. Rural	
103	Age of the mother/caretaker	______Years	
104	Marital status of the mother/caretaker	1. Currently Married<br>2. Divorced<br>3. Single<br>4. Widowed<br>5. Separated	
105	Family size of the household	1. <3<br>2. 3-5<br>3. 6-8<br>4. 9-11<br>5. >=12	
106	NumberofU5children	1. 1<br>2. 2<br>3. 3<br>4. >3	
107	Ethnic group of parents/caretakers	1. Oromo<br>2. Amhara<br>3. Others(Specify):	

108	Educational level of mother/caretaker	1. Formal education (A. Primary, B. Secondary, C. Higher)<br>2. Read and write<br>3. Read only<br>4. Neither write nor read	
109	Occupation of mother/caretaker	1. Housewife<br>2. Government employee<br>3. Merchant<br>4. Farmer<br>5. Other(specify):	
110	Age of the child's father	Years	
111	Educational level of the father	1.Formal education (last grade completed)<br>2.Read and write<br>3.Readonly<br>4.Neither	
112	Occupation of the father	1.Governmentemployee<br>2.Merchant<br>3.Farmer<br>4.Nojob<br>5.Other(specify	
113	Religion	1. Muslim<br>2. Orthodox Christian<br>3. Others(specify): ____	
114	Monthly income of the household	1. <500birr<br>2. 501-1000birr<br>3. >1000birr<br>4. Do not know(unspecified)	
**PARTII. HOUSEHOLDENVIRONMENTALHEALTHCONDITIONS**			
201	Do you have latrine facility?	1.Yes<br>2.No →	207

202	Ownership of the latrine	1.Privately owned<br>2.Shared with neighbors	
203	Type of latrine facility (observation)	1. Traditional pit latrine<br>2. VIP latrine<br>3. Public latrine<br>4. Communal latrine<br>5. Other(specify):	
204	Does the latrine currently functioning?	1. Yes<br>2. No	
205	Is there a place for washing hands? (observe)	1. Yes<br>2. No	
206	Is there a soap/ash in a place they wash their hands? (observe)	1. Yes<br>2. No	
207	If the family has no latrine, where do you dispose Human waste (Adult member open defecation)	1. Open field<br>2. Other(specify	
208	Is feces seen around the house (or in the compound)?	1. Yes<br>**2.** No	
209	How do you dispose refuse? **(Multiple response is possible)**	A. Pit<br>B. Open field<br>C. Burning<br>D. Garbage can<br>E. Other(specify**):**	
210	Where you dispose infant feces/excreta?	1. Not in latrine<br>2. In latrine	
211	What is the main source of drinking water for members of your household	1. Piped water<br>2. Water from open well<br>3. Water from borehole<br>4. Surface water	
212	Daily water consumption (Per capita water consumption)	1. ≤20Liters<br>2. >20Liters	
213	Distance from the house to the water source	Minutes	
214	Is water available all the times?	1. Yes	

		2. No	
215	Number of rooms in the house	1. 1<br>2. 2<br>3. 3andabove	
216	Are there domestic animals living in the same house with the members of the family?	1. Yes<br>2. No	
**PARTIII: BEHAVIORALASPECTS**			
301	When do you wash your hands?	1. Before food preparation and eating<br>2. After eating<br>3. After visiting latrine<br>4. After cleaning of child bottom<br>5. Other (specify):	
302	By using what do you wash your hands?	1. Soap & water<br>2. Ash & water<br>3. Only water<br>4. Other (specify):	
303	Do you separately prepare food for the child, using a Separate material?	1.Yes<br>2.No	
304	What do you use to feed the child?	1. Hand<br>2. Cup and spoon<br>3. Cup<br>4. Bottle<br>5. Other(specify):	
305	Does the drinking-water storage container have a cover? Ask the respondent to show you the storage container	1.Yes<br>2.No	
306	Is there a separate can for taking drinking water from the storage container? Ask the respondent to	1.Yes<br>2.No	

	show you the Can		
307	Type of collection container	1.Pot<br>2.Plastic bucket<br>3.Iron bucket<br>4.Jerry can<br>5.Other (specify):	
308	Method of drawing of water from the storage container		
	Dipping	1. Yes<br>2. No	
	Pouring	1. Yes<br>2. No	
	Others(specify)…..		
309	Covering material is used during transportation	1. Yes<br>2. No	
310	Frequency of washing collection containers and others per week	1. Not washed<br>2. Once<br>3. Twice<br>4. ≥Three	
311	When is strict care while handling water?		
	Fetching	1. Yes<br>2. No	
	Transporting	1. Yes<br>2. No	
	Storing	1. Yes<br>2. No	
	Drinking	1. Yes<br>2. No	
	Others		
312	Do you treat your water in any way to make it safer to drink?	1. Yes<br>2. No	
313	What do you usually do to the	1. Boil	

	water to make it safer to drink?	2. Chlorine<br>3. Strain through cloth(Filtering)<br>4. Other(specify):______	
314	Why do you use this method for making water safer?	1. Cost<br>2. I don't know other option<br>3. The method is effective<br>4. I don't know<br>5. Others______________	
315	Why don't you treat your drinking Water?	1. Availability<br>2. Costs<br>3. Bad taste and smelly of treated water<br>4. I believe water is safe from the source<br>5. I am used to drink untreated, nothing happens to us<br>6. I don't know<br>7. Other:_______________	
316	Do you store water for drinking separately from water for other Domestic purposes?	1. Always<br>2. Sometimes<br>3. Never	
317	Which container do you use to store water for drinking? (observe and write answers)	1. Bucket with a lid<br>2. Bucket without a lid<br>3. Small pans<br>4. Jerry cans<br>5. Other:_______________	
318	How do you draw water from your Container?	1. Use small pan<br>2. Pour directly from container<br>3. Use cup<br>4. Other:_______________	

319	What causes diarrhea? (Do not read choices to the respondent)	1. Drinking dirty water<br>2. Eating contaminated food<br>3. Flies/Insects<br>4. Poor hygiene<br>5. Weather condition<br>6. Other:_____________	
320	How can you prevent diarrhea? (Don't read choices to respondent)	1. Wash hands more frequently<br>2. Cooking thoroughly<br>3. Cover prepared food<br>4. Cleanliness (dishes, utensils)<br>5. Other______________<br>6. Don't Know	
**PARTIV:INFORMATIONOF THECHILD**			
401	Age of the child (in Months)	1. 0-5Months<br>2. 6-11<br>3. 12-23<br>4. 24-35<br>5. 36-47<br>6. 48-59	
402	Sex of the child	1. Male<br>2. Female	
403	Birth order of the child	1. First<br>2. Second<br>3. Third<br>4. Fourth & above	
404	Nutritional status of the child (Observe)	1. Malnourished<br>2. Well nourished	
405	Do you (the mother/caretaker) have a history of diarrhea in	1.Yes<br>2.No	

	the past two weeks?		
406	Does this child on breast feeding or breastfed for 2 years before stop?	1. Yes<br>2. No →	407
407	For how long did you breastfed your child?	1. <1Year<br>2. ≥1 Years	
408	What is his/her current breastfeeding status?	1. Exclusive breastfeeding<br>2. Partial breastfeeding<br>3. Not breastfeeding	
409	At what age the child started supplementary /weaning food?	1. Before6Months<br>2. At6 Months<br>3. After6Months	
410	Did the child receive vaccination?	1. Yes<br>2. No	
411	Do your child have diarrhea in the past two weeks (Past 14 days)?	1. Yes<br>2. No →	End
412	For how long the diarrhea last?	1. Lessthan14days<br>2. Greaterthan14days	
413	If the child has diarrhea today, how many times a day he/she passes stool?	1. Three times<br>2. Less than three times<br>3. More than three times<br>4. Don't know	
414	The type of diarrhea that the child had	1. Watery<br>2. Blood and mucus	

Annex3: Afan Oromo Version questionnaires

Maqaa Gandaa Maqaa gooxii________________________

Koodii Lakk.Manaa______________

Maqaa nama raga funaanee_________________________Maallattoo_________

KUTAA TOKKOFFAA: HAALA WALIIGALAA

**Koodii**	**Gaaffilee**	**Deebii**	**Gara**
101	Walitti dhufeenya namni deebii kenne daa'ima waliin qabdu (Daa'imaaf maal taata?)	1. Abbaa<br>2. Haadha<br>3. Akaakayyuu<br>4. Akkawoo<br>5. Obboleessa/Obboleettii<br>6. Kanbiroo	
102	Teessoo maatii	1. Magaalaa<br>2. Baadiyyaa	
103	Umrii haadhaa	Waggaa___	
104	Haala gaa'ilaa haadhaa	1. Yeroo ammaa kan heerumte<br>2. Kan wal hiikte<br>3. Kan hin heerumne<br>4. Kan abbaan manaa irraa du'e<br>5. Gargar kan jiraatan	
105	Baay'inni maatii meeqa?	1. <3<br>2. 3-5<br>3. 6-8<br>4. 9-11<br>5. >=12	
106	Baay'ina daa'imman waggaa 5 gadii	1. 1	

		2. 2<br>3. 3<br>**4. >3**	
107	Sabni kee maali?	1. Oromoo<br>2. Amaara<br>3. Kan biroo (ibsi):_____	
108	Sadarkaa barumsaa haadhaa/guddistuu daa’imaa	1. Barnoota idilee (**A**. Sad.1ffa xumure, **B.** Sad.2ffaa xumure,**C.**Sad.olaanaa)<br>2. Dubbisuu fi barreessuu<br>3. Dubbisuu qofa<br>4. Barreessuus dubbisuus hin danda’u	
109	Hojii haadhaa/guddistuu daa’imaa	1. Haadha manaa<br>2. Hojjattuu mootummaa<br>3. Daldaltuu<br>4. Qotee bulaa<br>5. Kan biroo (ibsi):______	
110	Umuriin Abbaa daa’imaa meeqa?	Waggaa_____	
111	Sadarkaa barumsa abbaa daa’imaa	1. Barnoota idilee (**A**. Sad.1ffaxumure, **B.** Sad.2ffaa xumure, C. Sad.3ffaa)<br>2. Dubbisuu fi barreessuu ni danda’a<br>3. Dubbisuu qofa danda’a<br>4. Barreessuus dubbisuus hin danda’u	
112	Hojii abbaa daa’imaa	1. Hojjataa mootummaa<br>2. Daldalaa<br>3. Qotee bulaa<br>4. Hoji-dhabaa<br>5.Kan biro (ibsi): _____________	
113	Amantii	1. Musliima<br>2. Kiristiyaana Orthodoksii	

		3. Kan biraa (ibsi):_________	
114	Galii ji’aa	1. Qarshii 500 gadi<br>2. Qarshii 501-1000<br>3. Qarshii 1000 ol<br>4. Hin beeku (hin ibsamne)	
**KUTAA LAMMAFFAA: HAALA QULQULLINA NAANNOO**			
201	Mana fincaanii qabduu?	1. Eeyyee<br>2. Miti (Hin jiru) ⟶	207
202	Abbummaan mana fincaanii kan eenyuuti?	1. Kan dhuunfaati<br>2. Ollaadhaan walitti fayyadamna	
203	Gosa mana fincaanii kami (Ilaali)	1. Mana fincaanii kan aadaa<br>2. Mana fincaani fooyya’aa<br>3. Mana fincaanii kanu mmataa<br>4. Mana fincaanii kan waliinii<br>5. Kan biro (ibsi)	
204	Amma manni fincaanii tajaajila kennaa?	1. Eeyyee<br>2. Miti	
205	Bakki harka dhiqannaa mana fincaanii bira jiraa?	1. Eeyyee<br>2. Miti (Hin mul’atu)	
206	Saamunaan/daaraan bakka harka dhiqannaa jiraa?	1. Eeyyee<br>2. Miti (Hin mul’atu)	
207	Mana ficaanii yoo hin qabanne, balfa namaarra bahu eessatti gattu? (Nama ga’eessa taheef)	1. Dirree gubbatti<br>2. Kan biroo (ibsi):_	
208	Sagaraan/booliin naannawa manatti/dallaa keessatti ni mul’ataa?	1.Eeyyee<br>2. Miti (Hin mul’atu)	
209	Akkamitti balfa/kosii mana keessaa bahu gattu? (Deebiin tokkoo ol ni danda’ama)	A. Boolla<br>B. Dirree gubbatti<br>C. Gubuu<br>D. Kan biraa (ibsi):______	
210	Sagaraa/boolii daa’immanii eessatti gattu?	1. Mana fincaaniitiin alatti<br>2. Mana fincaanii keessatti	
211	Maddi bishaan dhugaatii keessanii	1. Bishaan sararaa	

	eessa?	2. Burqaa<br>3. Bishaan boollaa<br>4. Bishaan lagaa	
212	Guyyaatti bishaan liitira meeqatti fayyadamtu?	1. ≤20L<br>2. >20L	
213	Fageenyi maddi bishaanii manarraa nama deemsisu yeroodhaan yoo ibsamu meeqa?	Daqiiqaa________	
214	Yeroo hunda bishaan nia rgattuu?	1. Eyyee<br>2. Lakki	
215	Manni jireenyaa gola meeqa qaba?	1. 1<br>2. 2<br>3. 3 fi Sanaa ol	
216	Miseensa maatii waliin horiin mana jiraatu jiraa?(Ilaali)	1. Eeyyee<br>2. Miti	
**KUTAA SADAFFAA: GOCHA/AMALA**			
301	Yeroo kam harka dhiqattu?	1. Nyaata qopheessuu fi nyaachuun dura<br>2. Nyaata booda<br>3. Mana fincaanii booda<br>4. Daa'ima erga qulqulleesse booda<br>5. Kan biro (ibsi):____	
302	Harka maaliin dhiqattu?	1. Saamunaa fi bishaaniin<br>2. Daaraa fi bishaaniin<br>3. Bishaan qofaan<br>4. Kan biro (ibsi):__	
303	Nyaata daa'imaa meeshaa Qophaatti fayyadamuun qophaatti qopheessituu?	1. Eeyyee<br>2. Miti	
304	Daa'ima maal fayyadamuun nyaachifta?	1. Harkaan<br>2. Kubbaayyaa fi fallaana<br>3. Kubbaayyaa<br>4. Qaruuraa	

		5. Kan biro(ibsi):	
305	Bishaan dhugaatiif kuufame qadaadamaa? (ILAALI)	1. Eeyyee<br>2. Miti	
306	Bishaan meeshaa itti kuufame keessaa baasuuf/waraabuuf Oolu meeshaan qophaa jiraa?	1. Eeyyee<br>2. Miti	
307	Bishaan meeshaa kamiin waraabbattu?	1. Hubboo/Okkotee<br>2. Baaldii pilaastikaa<br>3. Baaldii sibiilaa<br>4. Jaarikaana<br>5. kan biroo	
308	Mala bishaan meeshaa itti kuufame keessaa baasan		
	Keessa kaa'uun	1. Eeyyee<br>2. Miti	
	Gadi jallisuun	1. Eeyyee<br>2. Miti	
	Kan biroo (ibsi)		
309	Meeshaan bishaan ittin waraabbatan yeroo karaa deemtaniin ni qadaadama?	1. Eeyee<br>2. Miti	
310	Meeshaa bishaan ittiin waraabbattan torbaanitti si'a meeqa dhiiqxu?	1. Hin dhiqamu<br>2. Si'a tokko<br>3. Si'a lama<br>4. Si'a sadii fi isaa ol	
311	Eeggumsa cimaa bishaaniif yeroo kam keennitu?		
	Yeroo Waraabbii	1. Eeyyee<br>2. Miti	
	Yeroo karaa deemu	1. Eeyyee<br>2. Miti	
	Yeroo kuufamu	1. Eeyyee<br>2. Miti	
	Yeroo dhugaati	1. Eeyyee	

		2. Miti	
	Kan biroo (ibsi)		
312	Bishaan dhugaatii dura wal'aantuu?	1. Eeyyee<br>2. Miti	
313	Bishaan dhugaatii qulqulleessuuf maal gootu?	1. Danfisuu<br>2. Kiloorina itti makuu<br>3. Dhimbiibuu<br>5. Kan biro (ibsi):______	
314	Mala kana maaliif fayyadamtu?	1. Gatii<br>2. Mala biro hin beeknu<br>3. Malichi bu'a qabeessa<br>4. Hin beeku<br>5. Kan biro____________________	
315	Bishaan dhugaatii keessan maaliif hin qulqulleessitan?	1. Hin jiru<br>2. Gatii<br>3. Foolii fi dhandhama jijjiira<br>4. Madda isaa irraa qulqulluu waan ta'eef<br>5. Osoo hin qulqulleessiin dhugnee omaa nu hin godhu<br>6. Hin beeku<br>7. Kan biro_____________________	
316	Bishaan dhugaatii bishaan tajaajila birootiif oolu irraa adda baastanii olkeessuu?	1. Yeroo hunda<br>2. Yeroo tokko tokko<br>3. Gonkumayyuu	
317	Bishaan dhugaatii maalitti kuustu?	1. Baaldii qadaada qabu<br>2. Baaldii qadaada hin qabne<br>3. Xaasaa xixiqqoo<br>4. Jerikaana<br>5. Kan biro_________________	
318	Bishaan akkamitti buufattuu?	1. Xaasaa xiqqoo fayyadamuun<br>2. Jallisnee buusna<br>3. Kubbaayyaa fayyadamuun<br>4. Kan biro_________________	

319	Dhibee garaa kaasaa maaltu fida? (Filannoo hin dubbisiiniif)	1. Bishaan qulqulluu hin taane dhuguu<br>2. Nyaata faalame nyaachuu<br>3. Titiisa/Ilbiisota<br>4. Qulqullina dhabuu<br>5. Haala qilleensaa<br>6. Kan biro_______	
320	Dhibee garaa kaasaa akkamitti ittisuun danda'ama? (Filannoo hin dubbisiiniif)	1. Yeroo murteessoo ta'anitti harka dhiqachuu<br>2. Nyaata sirriitti bilcheessuu<br>3. Nyaata bilchaate qadaaduu<br>4. Meeshaalee nyaataa qulqulleessuu<br>5. Kan biroo----------<br>6. Hin beeku	
**KUTAA AFRAFFAA: ODEEFFANNOO WAA'EE DAA'IMAA**			
401	Umriin daaimaa meeqa (Ji'aan)	1. 0-5<br>2. 6-11<br>3. 12-23<br>4. 24-35<br>5. 36-47<br>6. 48-59	
402	Saala daa'imaa	1. Dhiira<br>2. Dhalaa	
403	Ilmoo/ilma meeqaffaa dhalatee?	1. Angafa<br>2. 2ffaa →<br>3. 3ffaa<br>4. 4ffaa & sanaa ol	
404	Haala sirna soorataa daa'imaa	1. Sirriitti kan soorame<br>2. Sirriitti kan hin sooramne	
405	Torbaan lamaan darbe keessatti haadha/guddistuu daa'imaa dhibeen garaa kaasaa qabee beeka?	1. Eeyyee<br>2. Miti	

→

406	Daa’imni kun harma osoo hin guusiin waggaa lamaaf harma hodhee jiraa/yeroo ammaa harma hodhaa jiraa?	1. Eeyyee<br>2. Miti ⟶	407
407	Yeroo hangamiif daa’ima harma hoosifte?	1. <1Waggaa<br>2. ≥1Waggaa	
408	Yeroo amma sadarkaan harma hoosisaa daa’ima kami?	1. Harma qofa luuga<br>2. Makaa/nyaataa fi harma<br>3. Harma luuguu dhaabe	
409	Daa’imni nyaata dabalataa fuudhachuu yoom eegale?	1. Ji’a 6 dura<br>2. Ji’a 6tti<br>3. Ji’a 6 booda	
410	Daa’imni talaallii fudhatee jiraa?	1. Eeyyee<br>2. Miti	
411	Torbaan lamaan darbe keessatti (Guyyaa 14 darbe keessatti) daa’ima garaa kaasaan qabee jiraa?	1. Eeyyee<br>2. Miti ⟶	End
412	Guyyaa meeqaaf garaa kaasaan irra ture?	1. Guyyaa<14<br>2. Guyyaa>14	
413	Guyyaatti si’a meeqa garaan baasa?	1. Si’a 3<br>2. Si’a 3 gadi<br>3. Si’a 3 ol<br>4. Hin beeku	
214	Gosa garaa kaasaa akkamiitu irra ture?	1. Dhangala’aa akka bishaanii<br>2. Dhiigaan wal makaa	
